# Impact of vaccination status on clinical outcomes of hospitalized COVID-19 patients

**DOI:** 10.1186/s12879-024-09139-w

**Published:** 2024-02-23

**Authors:** Mohsen Gholinataj Jelodar, Samaneh Mirzaei, Fatemeh Saghafi, Shahab Rafieian, Soheil Rezaei, Alireza Saatchi, Ziba Dehghani Avare, Mahdie Dehghan Niri

**Affiliations:** 1 Clinical Research Development Center, School of Medicine, Shahid Rahnemoon Hospital, Shahid Sadoughi University of Medical Sciences, Yazd, Iran; 2 Division of Pulmonary and Critical Care Medicine, Department of Internal Medicine, School of Medicine, Shahid Sadoughi University of Medical Sciences, Yazd, Iran; 3 Department of Health in Emergencies and Disasters, School of Public Health, Shahid Sadoughi University of Medical Sciences, Yazd, Iran; 4 Department of Clinical Pharmacy, School of Pharmacy and Pharmaceutical Sciences Research Center, Shahid Sadoughi University of Medical Sciences, Yazd, Iran; 5 Department of Thoracic Surgery, Imam Khomeini Hospital, Tehran University of Medical Sciences, Tehran, Iran

**Keywords:** Clinical outcomes, COVID-19, Vaccination, Hospitalized patients

## Abstract

**Introduction:**

It is important to identify the relationship between the COVID-19 vaccination status and the prognosis of this disease in hospitalized patients to gain a more accurate picture of their status and the effect of vaccination, as well as take necessary measures to improve their medical care. Thus, the present study was conducted to investigate the relationship between the vaccination status of hospitalized COVID-19 patients and the disease severity index in terms of clinical, imaging, and laboratory criteria.

**Methods:**

This research is a descriptive-analytical cross-sectional study. the study population consisted of patients with a positive RT-PCR test for coronavirus, admitted to COVID-19 departments of teaching hospitals in Yazd, Iran, during two months in the sixth peak of COVID-19. The patients’ data comprised demographic information (age, sex, and underlying disease), clinical information (length of hospital stay, length of ICU stay, and vaccination status), disease outcome (mortality and intubation), laboratory information (ESR, CRP, and NLR), and imaging information (lung involvement percentage), and finally, the relationship between patients’ vaccination status and disease severity indices were analyzed with the chi-square test, independent t-test, and logistic regression analysis at a 95% confidence interval (CI).

**Findings:**

According to research findings, the duration of hospitalization was 5.25 ± 2.34 and 6.11 ± 3.88 days in groups of patients with complete and incomplete vaccination, respectively (*P* = 0.003). The lengths of ICU stay were 6 ± 4.63 and 5.23 ± 3.73 days in both groups of patients admitted to the ICU (*P* = 0.395). Furthermore, there were significant relationships between the ICU admission rates, endotracheal intubation, mortality rate, the lung involvement score in the chest CT scan, and the NLR with the vaccination status.Multivariate regression analysis indicated that DM, IHD, NLR, CT scan score and vaccination status were related to patients’ in-hospital mortality.

**Conclusion:**

Complete vaccination of COVID-19 led to a milder disease in terms of clinical, imaging, and laboratory criteria of patients and decreased the possibility of hospitalization in ICUs, intubation, and mortality in patients.

## Introduction

During the COVID-19 pandemic, efforts to control the prevalence of the coronavirus by personal protections (decreasing interpersonal contact, washing hands, social distancing, and wearing masks) or national measures (school closures, travel restrictions, and quarantine) partially limited its transmission but did not change the basic principles of the pandemic [1]. Even though some drugs are used to treat patients with severe COVID-19 [2, 3], vaccination has been considered a promising prophylactic strategy and a key strategy to stop the COVID-19 pandemic [4, 5].

People are vaccinated with two general objectives in mind. The first is direct protection and reduction of infection in people at risk, and the second is indirect protection due to public vaccination in society and, in fact, decreasing the number of cases of infection and disease in society [6, 7]. Attention to two important features related to COVID-19 indicates the importance of vaccination in the population: First, since this virus spreads exponentially, any slight delay in vaccination can cause very different results. Second, the spread of this virus is “over-dispersed,” Therefore, many specialists, including the World Health Organization (WHO), agree that vaccination is the best method to reduce the transmission and effects of COVID-19 ( [8]) and provides national and global opportunities to control the pandemic [9].

Since the emergence of the COVID-19 pandemic, studies have begun to build a COVID-19 vaccine based on various platforms in different countries. Due to the failure of the herd immunity strategy to create immunity against COVID-19, efforts have been intensified to develop a COVID-19 vaccine since early 2020, and thus billions of doses of vaccine have been produced and injected based on various platforms. According to the results of public vaccination in different countries and the control of disease mortality, vaccination can be considered the main method of controlling this disease. Vaccines are probably effective against different strains of the virus because they induce broad immune responses. Studies indicate that completely vaccinated people, even at a much lower rate than unvaccinated people, still get infected with COVID-19, and no vaccine is 100% effective, but immunization widely decreases hospitalization and death risks [6, 8, 10–16].

Some vaccines have been effective in preventing hospitalization and decreasing mortality [16–18]. A CDC evaluation in 24 hospitals indicates that partial vaccination against COVID-19 was 64% effective against COVID-19 hospitalization among adults aged 65 and older and 94% effective in completely vaccinated adults aged 65 and older. Hence, sufficient efforts by the government to rapidly enhance vaccination among all eligible age groups can reduce the number of patients with COVID-19 and its severe outcomes [19]. Hospitalization and mortality rates due to COVID-19 are significantly higher in unvaccinated people than in vaccinated ones. A one-month report in England indicated that the hospitalization rate of people over 80 years of age was 50.5 per 100,000 in completely vaccinated people and 143.9 per 100,000 in unvaccinated people, but the mortality rates were 45.5 and 145.4 per 100,000 people, respectively [20]. It is worth noting that the impact of vaccination on mortality from COVID-19 is much faster than the impact on hospital and ICU admissions [21].

The results of the vaccine trial indicated that the vaccines can create great immunity in both previously infected and uninfected individuals, ranging from 92% for recorded infections, 87% for hospitalizations, and 92% for severe disease [22]. It is therefore crucial to identify the relationship between COVID-19 vaccination and the prognosis of this disease in hospitalized patients to create an accurate picture of the patient’s health status and the effects of vaccination. In addition, it can be beneficial to provide better medical care for these patients. To this end, the present study was conducted to investigate the relationship between the vaccination status of hospitalized COVID-19 patients and disease severity indices in terms of clinical, imaging, and laboratory criteria.

## Method

### Study design and setting

This research is a descriptive-analytical cross-sectional study. the study population consisted of patients with a positive RT-PCR test for coronavirus, admitted in the COVID-19 department of teaching hospitals affiliated with Yazd University of Medical Sciences during the first two months of Omicron peak (from February 20, 2022, to March20, 2022).

Demographic information included age, sex, and underlying disease, clinical information included length of hospital stay and ICU stay, disease outcome (mortality and intubation), and laboratory information included NLR, CRP, and ESR, and imaging information (score of pulmonary involvement severity) of patients were extracted from records.

### Data collection

The present study received an ethical code, IR.SSU.SRH.REC.1400.027, from the ethics committee of Yazd Shahid Dr. Rahnemoun Hospital. Data collection was performed by rereading the patients’ files and visiting the health department for the patient’s vaccination status information. The patient’s demographic information (age, sex, and underlying disease), clinical information (length of hospital stay, length of ICU stay), disease outcome (mortality and intubation), laboratory information (NLR, CRP, and ESR), and imaging information (score of pulmonary involvement severity) were extracted. A radiologist evaluated CT scan involvement based on the CT scan observation and lung involvement severity report. The scoring system defined by the Fleischner Society’s glossary was employed in this issue. Based on the percentage of involvement, the scoring system assigns a value between 0 and 5 to evaluate lung lobe involvement. A score of 0 is given for no involvement, while a score of 1 is given for up to 5% lobar involvement. A score of 2 is assigned to lobar involvement ranging from 5 to 25%, whereas a score of 3 is given for involvement ranging from 25 to 50%. A score of 4 is given for lobar involvement ranging from 50 to 75%, and a score of 5 is given for involvement exceeding 75% [23]. The overall score is determined by summing up the five lung lobe involvement scores. On this basis, lung involvement severity was qualitatively classified as mild (0–8), moderate (9–16), and severe (17–25) by the authors.

Data on vaccination included type of vaccination (inactive, viral vector, Recombinant proteins) and Vaccination status were extracted. Vaccination status during the admission included the following cases: (1) Non-vaccination: The vaccine was not injected. (2) Partial vaccination: receiving only one or two doses of vaccine more than six months after the injection of the second dose. (3) Complete vaccination: Patients, who received three or two vaccine doses within six months of the second dose.

### Statistical analysis

Data analysis was performed using SPSS 26. Descriptive statistics included the mean, variance, standard deviation, and frequency, and inferential statistics included the chi-square test, independent t-test, and logistic regression analysis at a 95% confidence interval (CI). Chi-square and independent t-tests were used to check the correlation between quantitative and binary variables with vaccination status or mortality rate, respectively. Logistic regression was utilized to examine the relationship between the dependent variable (mortality rate) and the independent variables (age, NLR, underlying disease, length of ICU stay, length of hospital stay, and CT score). It is worth noting that the significance level was considered a *P*-value less than 0.05 in this study.

### Findings

The data of 615 patients with a positive PCR were analyzed. The mean age of patients was 58.06 ± 18.01, and 54.5% of them were females. Hypertension in 243 (39.5%) patients, diabetes in 188 (30.6%), and dyslipidemia in 143 (23.3%) were common underlying diseases in this study. The average duration of hospitalization for all patients (*n* = 615) in the hospital was 5.65 ± 3.17 days and the average duration of hospitalization in the ICU (*n* = 111) was 5.41 ± 3.94 days. In this study, 285 (53.7%) and 330 (46.3%) patientats were in the complete and incomplete vaccination groups, respectively. Table 1 presents other demographic, clinical, imaging, and vaccination data.

**Table 1:** Demographics and vaccination status of patients

General Data	*Overall (N = 615)*
**Sex, N (%)**	
Male	280(45.5)
Female	335(54.5)
**BMI, N (%)**	
< 30	425(69.1)
> 30	190(30.9)
**Comorbidities, N (%)**	
Hypertension	243(39.5)
Diabetes Mellitus	188(30.6)
Dyslipidemia	143(23.3)
Ischemic Heart Disease	97(15.8)
Chronic kidney disease	77(12.5)
Hypothyroidism	40(6.5)
Chronic Obstructive Pulmonary Disease	26(4.2)
Cancer	13(2.1)
Cerebrovascular accident	11(1.8)
**Imaging Result at Baseline**	
Score of lung involvement at baseline, N (%)	
0–8	439(71.4)
9–16	144(23.4)
17–25	32(5.2)
**Vaccination Status, N (%)**	
no vaccine	203(33)
Partial vaccination	127(20.65)
Complete vaccination	285(46.35)
**Vaccine type, N (%)**	
Inactive	341(55.44)
viral vector	52(8.5)
Recombinant proteins	3(0.5)
Recombinant proteins + inactive	12(2)
viral vector + inactive	4(0.7)
**Intubation, N (%)**	67(10.9)
**Death, N (%)**	82(13.3)

N: Number; BMI: Body Mass Index

According to the research findings, the intubation and mortality rates in the study were 67 (10.9%) and 82 (13.3%), respectively.

Table 2 presents the association between vaccination status and imaging, laboratory, and clinical information of the patients using the chi-square and independent t-tests. The duration of hospitalization was 5.25 ± 2.34 and 6.11 ± 3.88 days in the groups of patients with complete and incomplete vaccination, respectively (*P* = 0.003). The duration of ICU admission was 6 ± 4.63 and 5.23 ± 3.73 days in patients admitted to ICU in these two groups, respectively (*P* = 0.395).

As shown, there was a significant difference between the two groups in terms of ICU admission, endotracheal intubation, mortality rates, NLR, and the score of lung involvement in chest CT scans, with vaccination status.

**Table 2:** Association between vaccination status and characteristics of patients

Variable		vaccination		Total	*p*.value
		incomplete	complete		
hospital length of stay, day	Mean ± SD	6.11 ± 3.88	5.25 ± 2.34	615	0.001**
ICU length of stay, day	Mean ± SD	5.23 ± 3.73	6 ± 4.63	111	0.395**
ICU admission	N (%)	86(77.5)	25(22.5)	111	< 0.0001*
Death	N (%)	72 (25.3)	10(3)	82	< 0.0001*
Intubation	N (%)	57(20)	10(3)	67	< 0.0001*
CT Score	N (%)				
0–8 (mild)		156 (35.5)	283(64.5)	439	< 0.0001*
9–16 (Moderate)		105 (72.9)	39 (27.1)	144	
17–25 (severe)		24(75)	8 (25)	32	
CRP	N (%)				
Negative		63(22.10)	70(21.21)	133	0.658*
+		55(19.29)	71(21.51)	126	
2+		96(33.68)	98(29.69)	194	
3+		71(24.91)	91(27.57)	162	
NLR	Mean ± SD	7.92 ± 6.77	6.54 ± 5.95	615	0.007**
ESR	Mean ± SD	45.78 ± 30.34	46.14 ± 29.53	615	0.882**

N: Number, SD: Standard deviation, CRP: C Reactive protein, NLR: Neutrophil-Lymphocyte Ratio ESR: Erythrocyte Sedimentation Rate

*:Chi-square test, **: student’s *t*-test

Other findings of the present study included the comparison of mortality with imaging, clinical, and laboratory information with patients’ vaccination status using the chi-square and independent t-tests. In addition, underlying diseases, including diabetes, hypertension, dyslipidemia, and CVA showed significant differences. Furthermore, NLR, severity of lung involvement, vaccination status, length of hospital stay, the rate of admission to the ICU, and the rate of intubation had significant relationships with mortality rates. Table 3 presents these results.

**Table 3:** Association between demographic, clinical, paraclinical data, and vaccination status with patient mortality

variable		Death			*p*.value
		Yes		No	
Age	Mean(SD)	61.33(50.49)		57.55(17.57)	0.077**
Sex, N (%)	Male	32 (13.4)		248 (88.6)	0.204*
	Female	50 (14.9)		285 (85.1)	
BMI, N (%)	< 30	57 (10.4)		368 (86.6)	0.932*
	> 30	25 (13.4)		165 (86.6)	
Diabetes, N (%)	Yes	58 (30.9)		130 (69.1)	< 0.0001*
	No	40 (5.6)		403(64.4)	
Hypertension, N (%)	Yes	59(24.3)		184(75.7)	< 0.0001*
	No	23(6.2)		349(93.8)	
Ischemic heart disease, N (%)	Yes	37 (38.1)		60(61.9)	< 0.0001*
	No	45(8.7)		473(91.3)	
Dyslipidemia, N (%)	Yes	19 (13.3)		124 (86.7)	0.985*
	No	63 (13.3)		409 (86.7)	
CKD, N (%)	Yes	11(14.3)		66(85.7)	0.793*
	No	71(13.2)		467(86.8)	
COPD, N (%)	Yes	5 (19.2)		21 (80.8)	0.366*
	No	77 (13.1)		512 (86.9)	
Hypothyroidism, N (%)	Yes	7(17.5)		33 (82.5)	0.423*
	No	75 (13.0)		500 (87.0)	
CVA, N (%)	Yes	4 (36.4)		7 (63.6)	0.023*
	No	78 (12.9)		526 (87.1)	
Cancer, N (%)	Yes	3(23.1)		10(76.9)	0.296*
	No	79 (13.1)		523 (86.9)	
Vaccination status, N (%)	complete	10(3)		320(97)	< 0.0001*
	incomplete	72(25.3)		213(74.7)	
Score of lung involvement at baseline, N (%)	0–8	14 (3.2)		425(96.8)	< 0.0001*
	9–16	43(23.9)		101(70.8)	
	17–25	25(78.1)		7(21.8)	
CRP, N (%)	Negative	20(15)		113(85)	0.075*
	+	9(7.1)		117(92.9)	
	++	33(17)		161(83)	
	+++	20(12.3)		142(87.7)	
Intubation, N (%)	Yes	60(89.6)		7(10.4)	< 0.0001*
	No	22(4)		526(96)	
ICU admission, N (%)	Yes	62(56.4)		48(43.6)	< 0.0001*
	No	20(4)		485(96)	
Vaccine type, N (%)	Inactive	19(5.6)		322(94.4)	0.633*
	viral vector	5(9.6)		47(90.4)	
	Recombinant proteins	0		3(100)	
	Recombinant proteins + inactive	0		12(100)	
	viral vector + inactive	0		4(100)	
ESR		51.36(30.92)		45.15(29.66)	0.08**
	Mean(SD)				
NLR		13.37(8.53)		6.23(5.39)	< 0.0001**
ICU length of stay,y		5.75 (4.23)		4.96 (3.47)	0.30**
hospital length of stay,y		7.91(6.27)		5.30(2.18)	< 0.0001**

N: Number; BMI: Body Mass Index; y: year, SD:standard deviation, CKD: Chronic kidney disease, COPD: Chronic Obstructive Pulmonary Disease, CVA: Cerebrovascular accident, CRP:C Reactive protein, NLR: Neutrophil-Lymphocyte Ratio ESR: Erythrocyte Sedimentation Rate, ICU: Intensive Care Unit,

*:Chi-square test, **: student’s *t*-test

The study investigated the relationship between vaccination status and imaging, laboratory, and clinical information in patients with DM and IHD, due to their significant correlation with mortality rate. Based on their vaccination status, the two groups showed significant differences in ICU admission rates, duration of hospitalization, endotracheal intubation rates, and mortality rates. In diabetic patients, there is a significant relationship between vaccination status and the lung involvement score in the chest CT scan as well as the NLR. Similarly, in the group of cardiac patients, there is a significant relationship between vaccination status and the rate of admission to the ICU, mortality rate, intubation, and lung involvement scores in the chest CT scan. (Table 4).

**Table 4:** Association between vaccination status and characteristics of IHD and DM patients

Variable			DM patients				IHD patients			
			Vaccination				vaccination			
			incomplete	complete	Total	*p*.value	incomplete	complete	Total	*p*.value
**hospital length of stay**		Mean ± SD	6.79 ± 5.49	5.41 ± 2.14	188	0. 027**	6.36 ± 4.32	5.29 ± 2.50	97	0.134**
**ICU length of stay**		Mean ± SD	5.44 ± 4.12	4 ± 1.26	47	0.40**	5.52 ± 3.20	4.57 ± 0.78	30	0.448**
**ICU admit ion**		N (%)	41(87.2)	6(12.8)	47	< 0.0001*	21(75)	7(25)	30	< 0.0001*
**Death**		N (%)	51 (87.9)	7(12.1)	58	< 0.0001*	29(78.4)	8(21.6)	37	< 0.0001*
**intubation**		N (%)	32(86.5)	5(13.5)	37	< 0.0001*	18(75)	6(25)	24	0.001*
**CT Score**	**0–8 (mild)**	N(%)	47 (37.3)	79(62.7)	126	< 0.0001*	17(27.4)	45(72.6)	62	< 0.0001*
	**9–16 (Moderate)**		37 (82.2)	8 (17.8)	45		21(80.8)	5(19.2)	26	
	**17–25 (severe)**		14(82.4)	3 (17.6)	17		7(77.8)	2(22.2)	9	
**CRP**	**negative**	N (%)	27 (48.2)	29(51.8)	56	0.057*	11(52.4)	10(47.6)	21	0.278*
	**+**		18(62.1)	11(37.9)	29		9(50)	9(50)	18	
	**2+**		34(63)	20(37)	54		18(52.9)	16(47.1)	34	
	**3+**		19(38.8)	30(61.2)	49		7(29.2)	17(70.8)	24	
**NLR**		Mean ± SD	9.84 ± 8.21	6.80 ± 5.69	188	0.004**	7.54 ± 6.20	714 ± 5.27	97	0.733**
**ESR**		Mean ± SD	47.60 ± 33.45	44.21 ± 32.80	188	0.484**	47.35 ± 30.37	53.59 ± 33.54	97	0.342**

N: Number, SD: standard deviation, DM: Diabetes Mellitus, IHD: Ischemic heart disease, ICU: Intensive Care Unit

*:Chi-square test, **: student’s *t*-test

In order to identify the key factors that predict the mortality of patients who are hospitalized, we developed a model that takes into account the initial variables of patients upon arrival in the ICU. We included only those variables from Table 3 that had a *P*-value of less than 0.1, and subjected them to multivariate logistic regression analysis as part of our model-building process. The accuracy of the model fit was indicated by the Hosmer-Lemeshow test (*P* = 0.731). The final results of the model revealed that diabetes, vaccination status, ischemic heart disease, NLR, and CT score were effective predictors of mortality among patients with COVID-19. (Table 5).

**Table 5:** Determinants of mortality among the patients Based on primary variables

Variable	*p*. value	adjusted odds ratio (OR)	95% C.I. for OR	
			Lower	Upper
Vaccination status ^a^	< 0.0001	0.0760	0.0260	0.2170
Diabetes Mellitus	< 0.0001	9.28	3.56	24.18
Ischemic Heart Disease	< 0.0001	19.70	6.74	57.57
Hypertension	0.137	1.96	0.800	4.78
Cerebrovascular accident	0.6440	1.95	0.110	33.57
Erythrocyte Sedimentation Rate(ESR)	0.311	1.00	0.990	1.02
Neutrophil-Lymphocyte Ratio(NLR)	< 0.0001	1.14	1.08	1.21
CT score ^b^	< 0.0001	12.54	5.97	26.35
C-reactive protein(CRP)	0.4670	1.16	0.770	1.73
Age	0.3900	0.990	0.960	1.01
Constant	< 0.0001	0.0020		

The table shows all the variables: -2loglikelihood = 170.87; χ2 = 312.11, *p* < 0.0001. Hosmer-Lemeshow statistics = 5.24 with df = 8, *p* = 0.731. a: complete, b: Score of lung involvement: 17–25

## Discussion

The present study indicated a significant effect of vaccination on the clinical outcomes of hospitalized patients. there was a significant difference in ICU admission between the complete and incomplete vaccination groups and patients with incomplete vaccination had higher rates of endotracheal intubation, mortality, and length of hospital stay. According to the results, vaccinated patients had a lower in-hospital mortality rate than the opposite group (3% vs. 25.3%). In this regard, Rahman et al. (2022) compared the mortality rate of unvaccinated and vaccinated patients (11.17% vs. 1.53%) [12]. A study in India also reported that complete vaccination was correlated with a 70% reduction in the mortality rate of hospitalized COVID-19 patients, indicating the importance of the COVID-19 vaccination in reducing mortality, hospitalization, and other severe complications [22].

According to other results, patients with a history of incomplete vaccination during hospitalization needed more ICU admissions and had higher rates of endotracheal intubation, mechanical ventilation, and mortality. According to the findings of this study, intubation rates for completely to partially vaccinated patients were 3–20%. Other studies indicated that 4–4.7% of completely vaccinated patients needed mechanical ventilation, which was significantly different (16–18%) compared to groups without vaccination or with partial vaccination [24–26]. Comparing the clinical results of completely vaccinated people in other studies indicated that these patients required less supplemental oxygen and mechanical ventilation. They also had fewer hospitalizations, less severe diseases, and lower mortality rates, a result consistent with the present study [22, 24, 25, 27, 28]. Hence, vaccination provides significant protection for hospitalized patients. In confirmation of the present results, the reduction of ICU admission and the need for supplemental oxygen might be due to the IgG antibody response produced by vaccines that fight against SARS-CoV-2 strains, thereby reducing the severity of COVID-19-related disease [12]. COVID-19 vaccines boost the immune response against SARS-CoV-2 variants by inducing antibodies. Vaccines may also induce the body to produce T cells that destroy cells infected with COVID-19 and thus reduce COVID-19 mortality [29]. Regarding the number of vaccinations, there was no evidence for the long-term protection of COVID-19 by a single dose of the vaccine [30], and even the potential risk of delaying the second dose of the COVID-19 vaccine made it less effective. Therefore, it is suggested to receive the vaccine at the recommended interval. Some experts have warned that partial immunization with a single dose can lead to a lower immune response that provides only partial immunity against COVID-19 infection [31]. It is worth noting that a complete vaccination with two doses of the vaccine is an important factor in preventing COVID-19 morbidity and mortality, and the recommendation for the second dose of the vaccine from 4 to 8 weeks after the first dose is confirmed [31]. Nevertheless, although the overall effectiveness of the vaccine for the period of 7 days to 6 months after the second dose was more than 90%, as the time after the second dose increased, the effectiveness decreased. As it was shown in a study that after the second dose, the effectiveness of the vaccine against Covid-19 decreased to 90% within 2 to 4 months and to 84% within 4 to 6 months [32], and studies have also shown that the third dose increases immunity against SARS-CoV-2 [33–35]. These findings confirm the results of the present study about the effect of complete vaccination with three or two doses of vaccine under which the second dose is administerated less than six months after admission.

This study also indicated that patients with a history of incomplete vaccination had much more severe diseases in terms of imaging and laboratorydata. This high level of involvement resulted in significantly worse disease in patients. Therefore, patients with an incomplete vaccination or a lack of vaccination had a more severeinvolvement in the chest CT scan, and their NLR levels (an inflammatory factor of the hemogram) were significantly higher. Also, no significant difference was observed between other inflammatory indices such as CRP and ESR in the two groups. Various studies also indicated that the serum levels of CRP and NLR and the pulmonary involvement level in CT scans were correlated with the severity of COVID-19 and the patients’ prognosis [36–39]. The findings of this study confirmed this correlation with the mortality of patients except for CRP. The lack of quantitative data may have impacted the patient’s results in this issue since the serum CRP level was reported qualitatively.

Sagiraju et al. (2022) investigated the effect of vaccination on the inflammatory response. They reported an excessive inflammatory response with high levels of d-Dimer, IL-6, and CRP in cases without vaccination or with partial vaccination compared to completely vaccinated cases, indicating that vaccination decreased the risk of hypoxia and cytokine storm. However, when a patient had hypoxia and ARDS, the risk of severe morbidity and mortality was similar to unvaccinated individuals. It indicates that the inflammatory immune response pattern determines the clinical course, and outcome of COVID-19 [24]. On the contrary, less severe diseases and mortality were seen in our study in more critical patients admitted to ICU with complete vaccination compared to the opposite group.

Comorbidities, in addition to vaccination status, were important potential aspects that could affect the severity and prognosis of COVID-19. In this study, patients with a history of diabetes (OR = 9.28) and ischemic heart disease (OR = 19.70) had higher mortality rates, indicating the importance of underlying diseases in the prognosis of patients’ mortality from COVID-19. Albitar et al. (2020) reported that cases with five or more comorbidities were at a higher risk of death from COVID-19 than those without any comorbidities. Also, COVID-19 patients with a history of cardiovascular diseases (CVD) or chronic obstructive pulmonary diseases (COPD) had higher odds ratios of mortality [40]. These results were consistent with the present study about the correlation of cardiovascular diseases with the mortality rate. However, they were inconsistent in terms of COPD patients owing to the small number of chronic lung disease cases in the present study.

The results were consistent with a previous systematic review, indicating that comorbidities such as hypertension and diabetes increased the risk of mortality due to COVID-19 [41]. In this regard, Rahman et al. (2020) reported that unvaccinated COVID-19 patients with comorbidities were ten times more likely to die from complications of COVID-19 [12]. Hypertension and diabetes, as part of the metabolic syndrome, are considered an inflammatory context that exacerbates the rapid spread of cytokine storm that is associated with the severity of COVID-19 [42–44]. Subgroup statistical analysis was performed since cardiovascular diseases and diabetes are considered important risk factors for disease severity and mortality. However, their complete vaccination was associated with milder pulmonary involvement, as well as lower need for admission to the ICU, endotracheal intubation, and lower mortality.

Regardless of the vaccination status, the COVID-19 mortality rate of hospitalized patients was 13.3%, while it was 22–97% in various studies on the mortality of patients with COVID-19 [22, 45–48]. According to other findings of the present study, NLR (OR = 1.14), and lung involvement percentage (OR = 12.54) had a significant correlation with mortality. Previous studies have also confirmed the correlation of these factors with mortality rates [39, 49].

### Research limitations

The present study was limited to two medical centers in two months, and it could have been conducted in a larger and multicenter population for a more detailed examination over a longer period. Several inflammatory factors other than those in the study were not routinely measured in our hospital to determine inflammatory status, so not measuring other inflammatory factors may have affected the study results. Also, this study only focused on the duration of hospitalization, and the patients’ information after discharge from the hospital was not available to check the prognosis.

## Conclusion

The results of the present study indicated that complete vaccination has led to a decrease in ICU admission and a decrease in mortality, along with milder disease in terms of clinical, laboratory, and imaging criteria in hospitalized patients. Furthermore, vaccination had a positive effect on the reduction of mortality risk even in high-risk patients with underlying diseases (DM and IHD). In addition to vaccination status, other factors including diabetes, IHD, NLR and CT score were identified as predictors of mortality in hospitalized COVID-19 patients.

## Data Availability

The datasets generated and analyzed during the current study are not publicly available to protect the participants’ confidentiality. However, they are available from the corresponding author on reasonable request.
